# Supplementary material for: Construction of hierarchically porous metal–organic frameworks through linker labilization
Source: Nat Commun. 2017 May 25;8:15356. doi: 10.1038/ncomms15356 (PMC5458506; doi:10.1038/ncomms15356)
Supplement: Supplementary Information — Supplementary Figures, Supplementary Tables, Supplementary Methods and Supplementary References [file ncomms15356-s1.pdf]

Supplementary Information

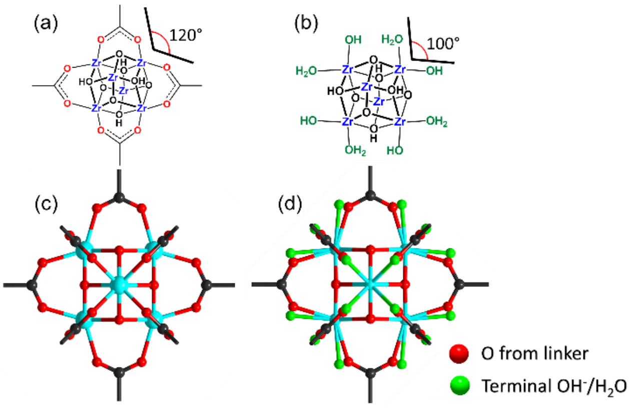

Supplementary Figure 1. Scheme representation of Zr_6 clusters terminated by carboxylates (a) and –OH/H_2O (b) highlighting different O−Zr−O bond angles; single-crystal structure of PCN-160-31% (left) and PCN-160-31%-2 (right), showing the emergence of terminal –OH/H_2O as an indication of defects.

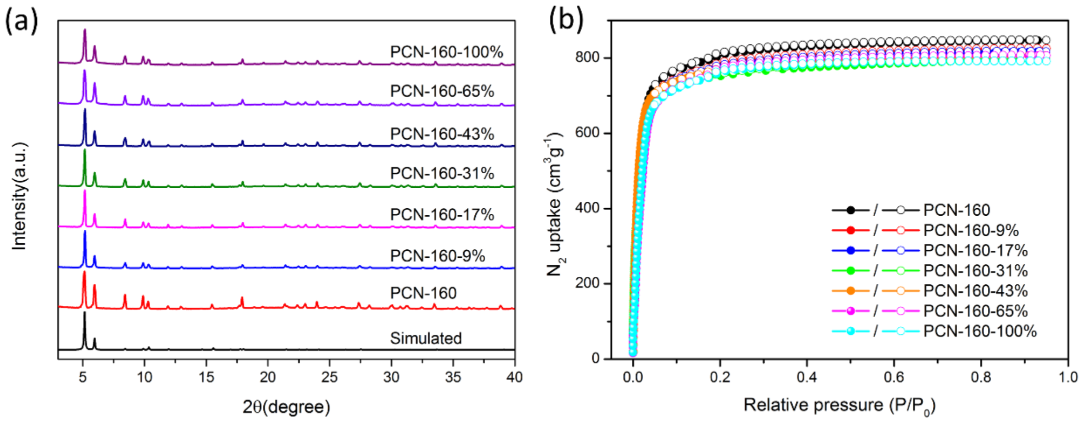

Supplementary Figure 2. PXRD patterns and N_2 sorption isotherms of PCN-160-R%.

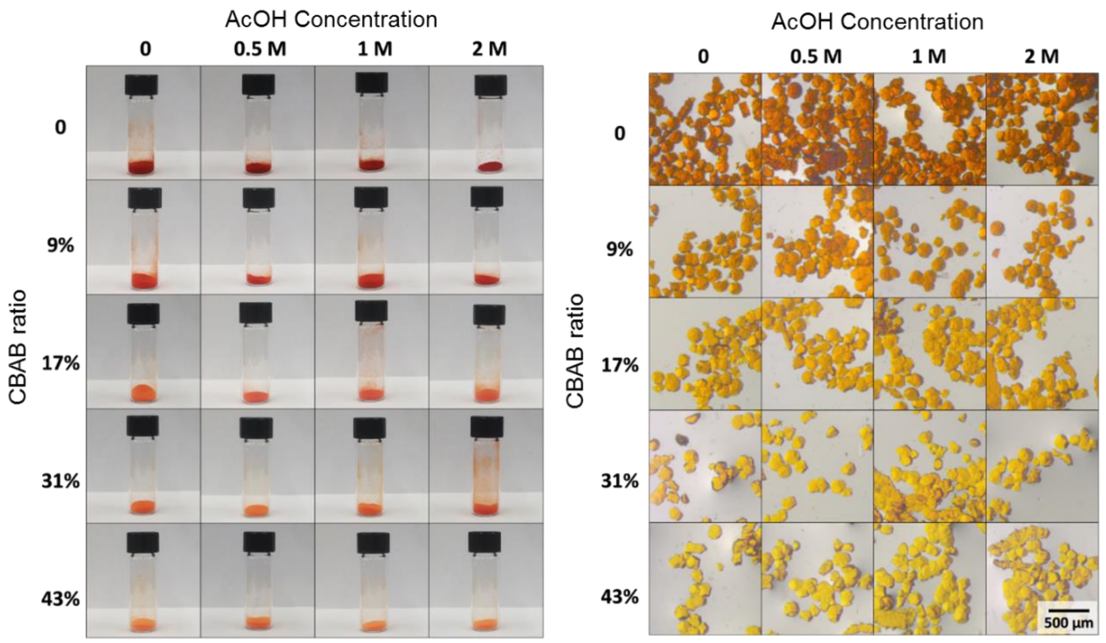

Supplementary Figure 3. Photographs and microscopic images of PCN-160-R%-*C* samples.

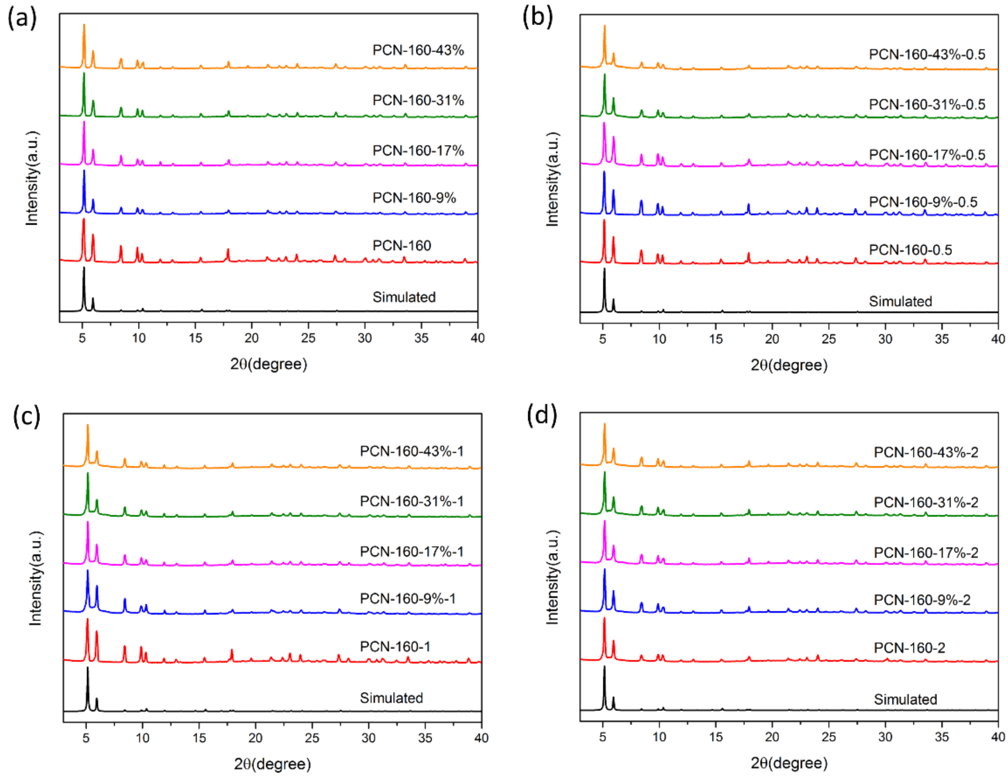

Supplementary Figure 4. PXRD patterns of PCN-160-R%-*C*.

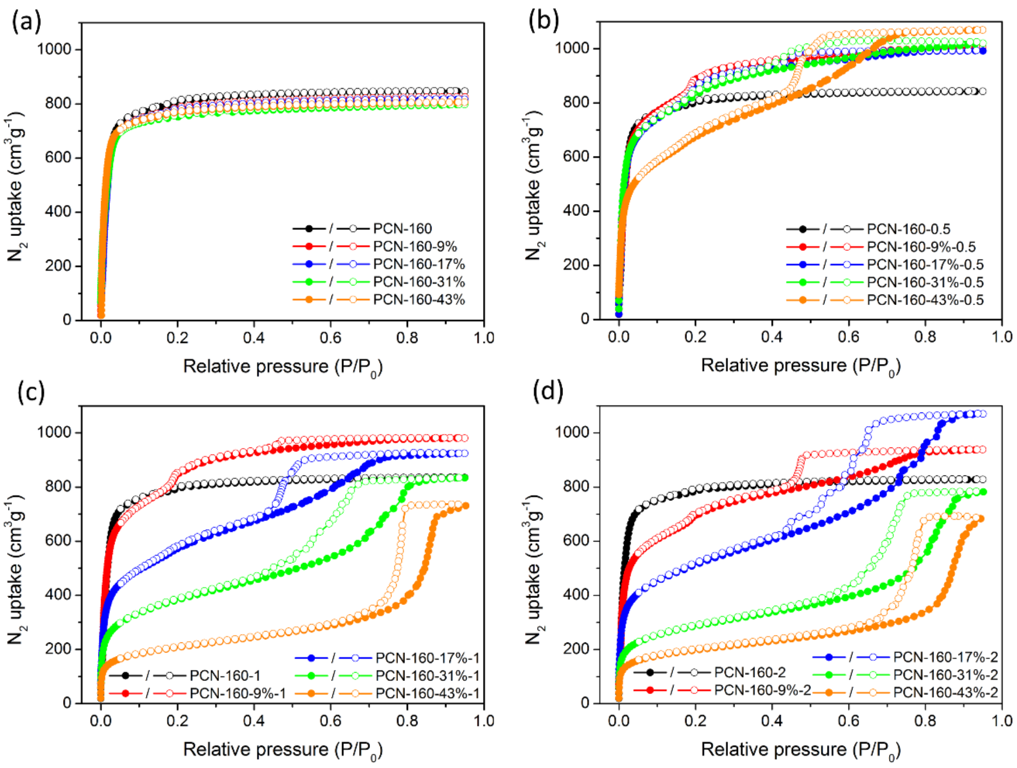

Supplementary Figure 5. N_2 sorption isotherms of PCN-160-R%-*C*.

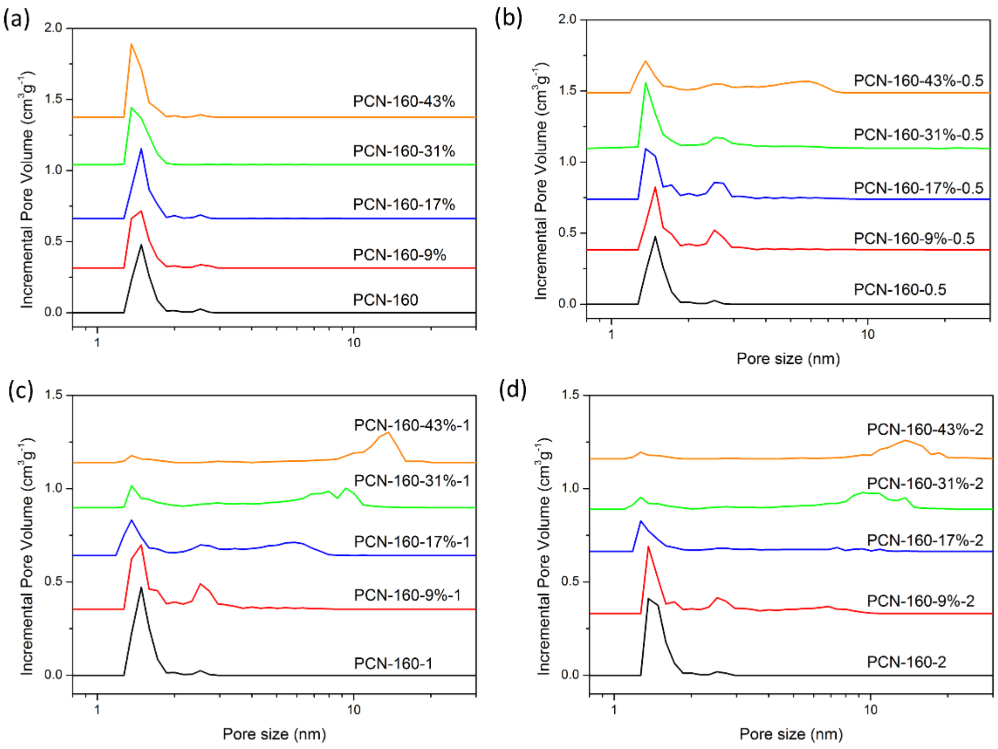

Supplementary Figure 6. Pore size distributions of PCN-160-R%-*C* calculated by density functional theory (DFT) method.

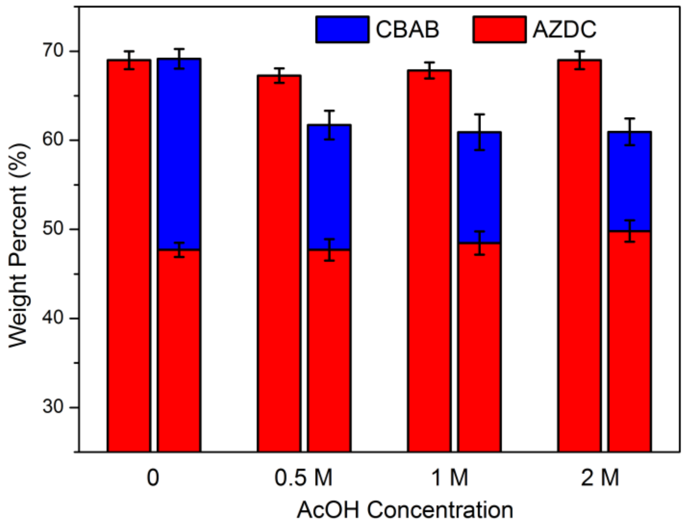

Supplementary Figure 7. Weight percent of CBAB and AZDC in PCN-160 and PCN-160-31% treated by different concentrations of AcOH.

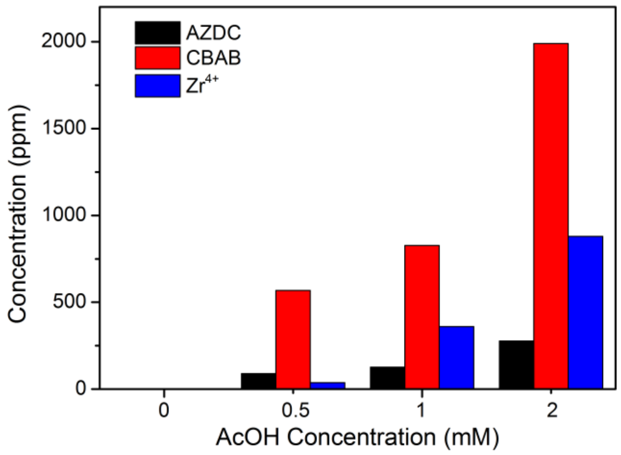

Supplementary Figure 8. Concentration of AZDC, CBAB and Zr^{4+} in the solution when PCN-160-31%-*C* was treated with different concentration of AcOH.

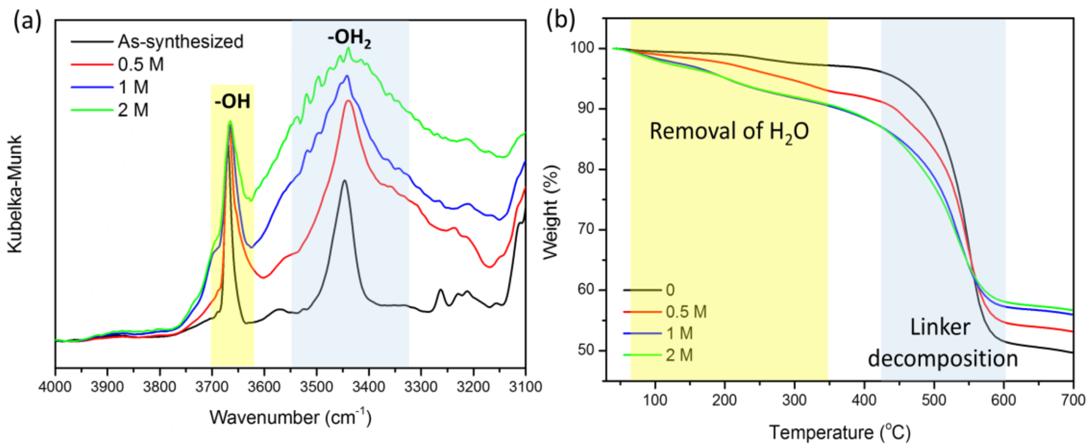

Supplementary Figure 9. (a) DRIFTS spectra and (b) TGA curves for PCN-160-31% treated with different concentration of AcOH.

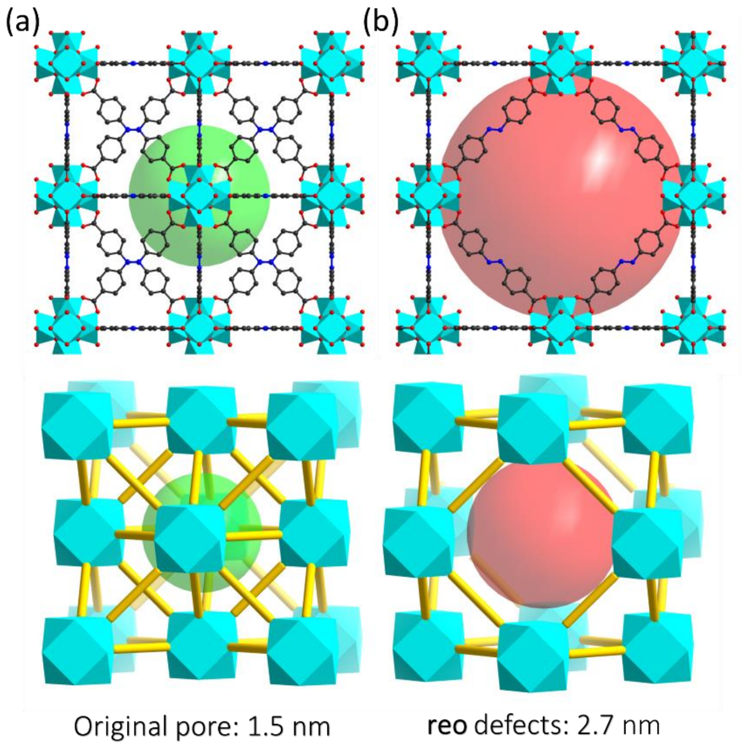

Supplementary Figure 10. (a) Structure and topology of PCN-160 with **fcu** topology; (b) Structure and topology of the **reo** defect region.

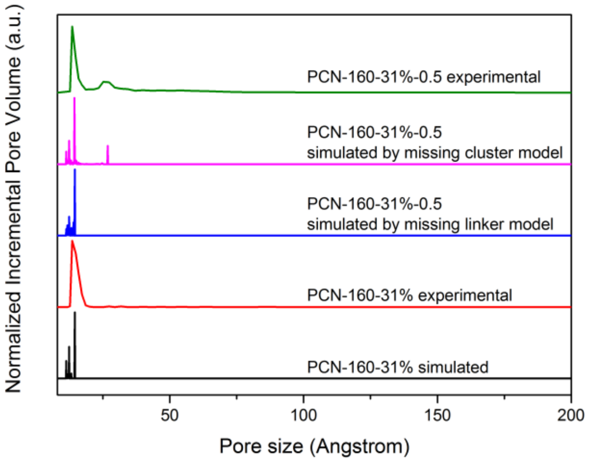

Supplementary Figure 11. A comparison of experimental and simulated pore size distribution based on missing-linker model and missing-cluster model.

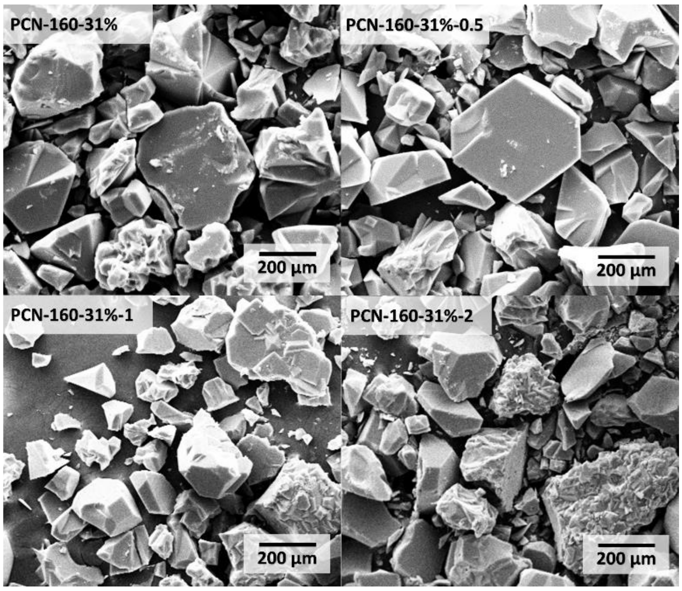

Supplementary Figure 12. SEM images of PCN-160-31% treated with different concentration of AcOH (scale bar 200 μm).

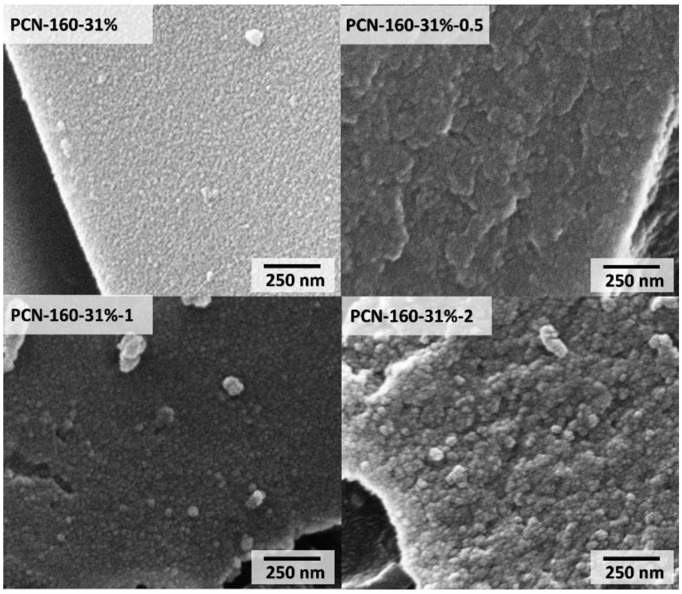

Supplementary Figure 13. SEM images of PCN-160-31% treated with different concentration of AcOH (scale bar 250 nm).

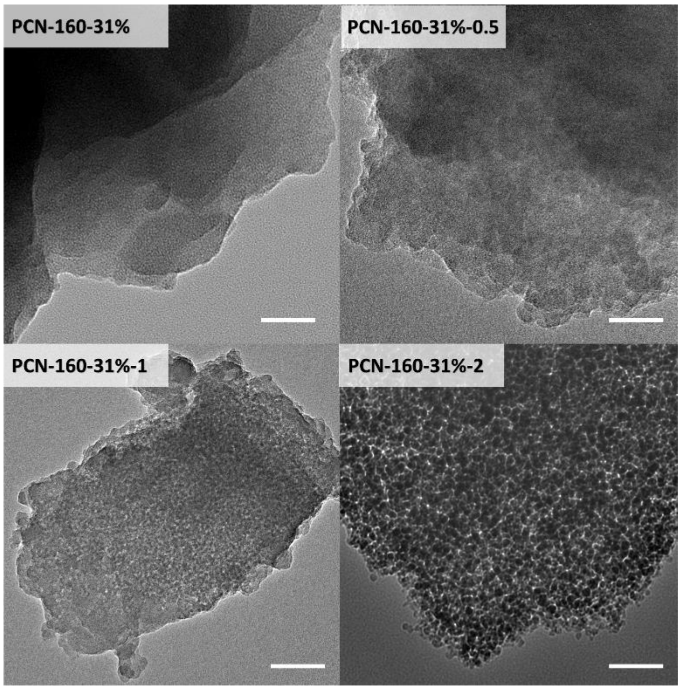

Supplementary Figure 14. TEM images of PCN-160-31% treated with different concentration of AcOH (scale bar 40 nm).

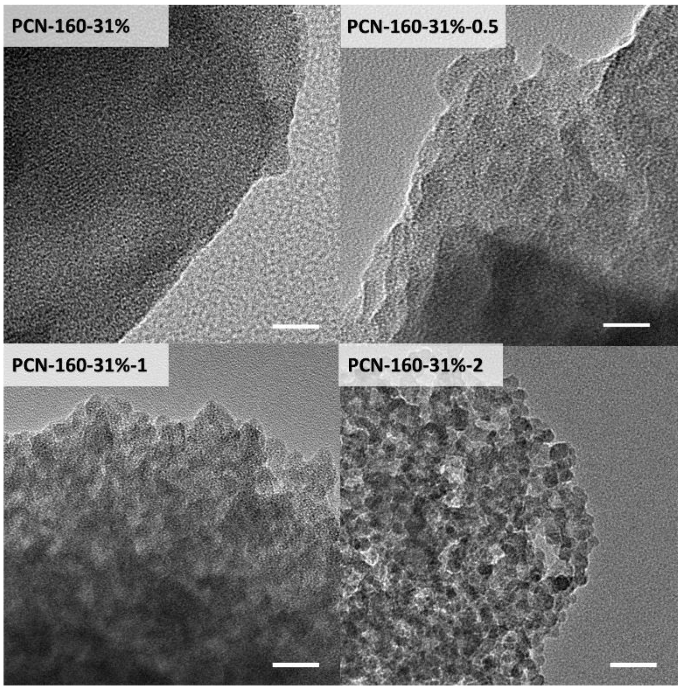

Supplementary Figure 15. TEM images of PCN-160-31% treated with different concentration of AcOH (scale bar 20 nm).

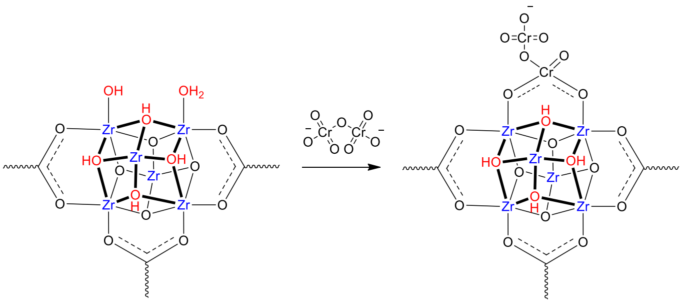

Supplementary Figure 16. Proposed adsorption mechanism of $Cr_2O_7^{2-}$ ion onto PCN-160-31%-*C* by coordinated at terminal $–OH^-/H_2O$ ligands.

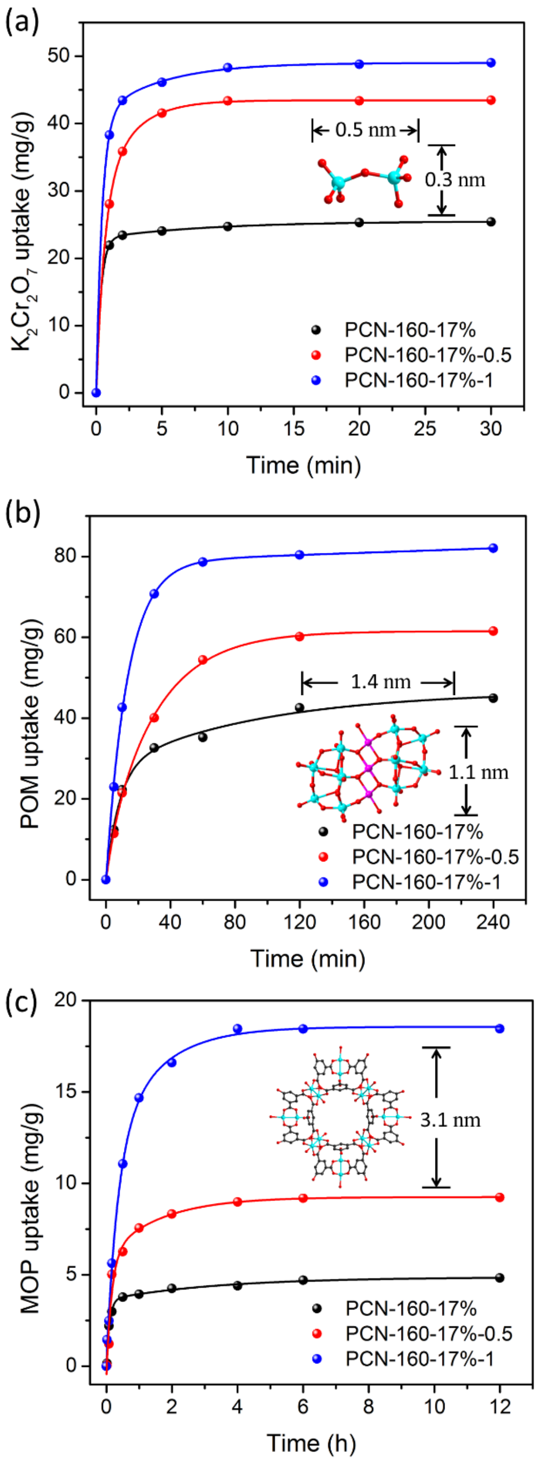

Supplementary Figure 17. Adsorption kinetics of $[Cr_2O_7]^{2-}$, POM and MOP in PCN-160-17%, PCN-160-17%-0.5, and PCN-160-17%-1, respectively.

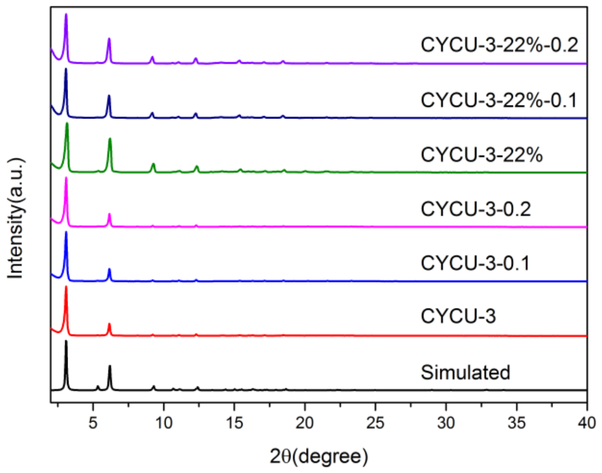

Supplementary Figure 18. PXRD patterns of CYCU-3 after different treatment.

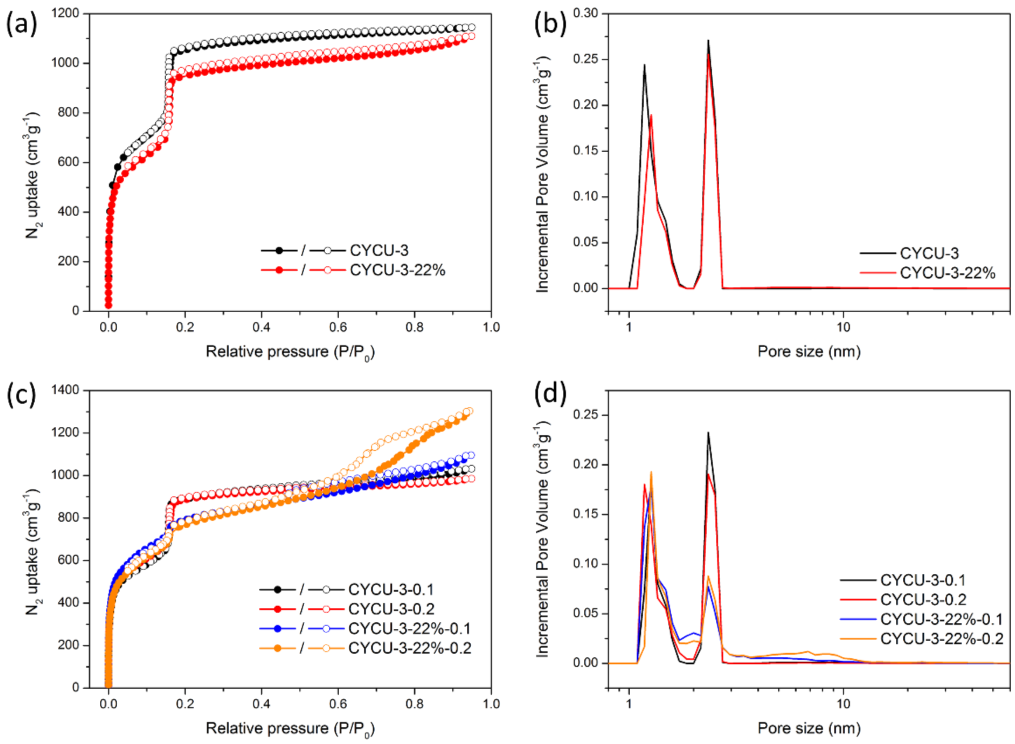

Supplementary Figure 19. N_2 sorption isotherms and pore size distributions of CYCU-3 after partial substitution of linkers (a),(b) and acid treatment (c),(d).

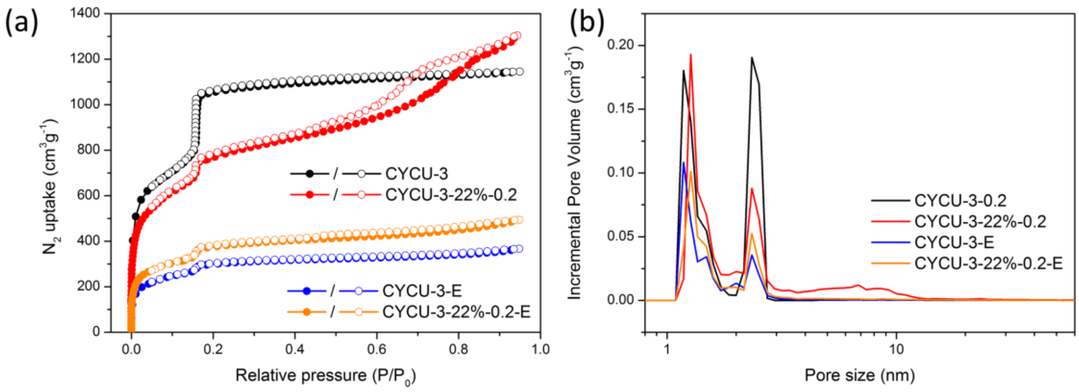

Supplementary Figure 20. N_2 sorption isotherms (a) and pore size distributions (b) of CYCU-3-22% and CYCU-3-22%-0.2 before and after enzyme loading.

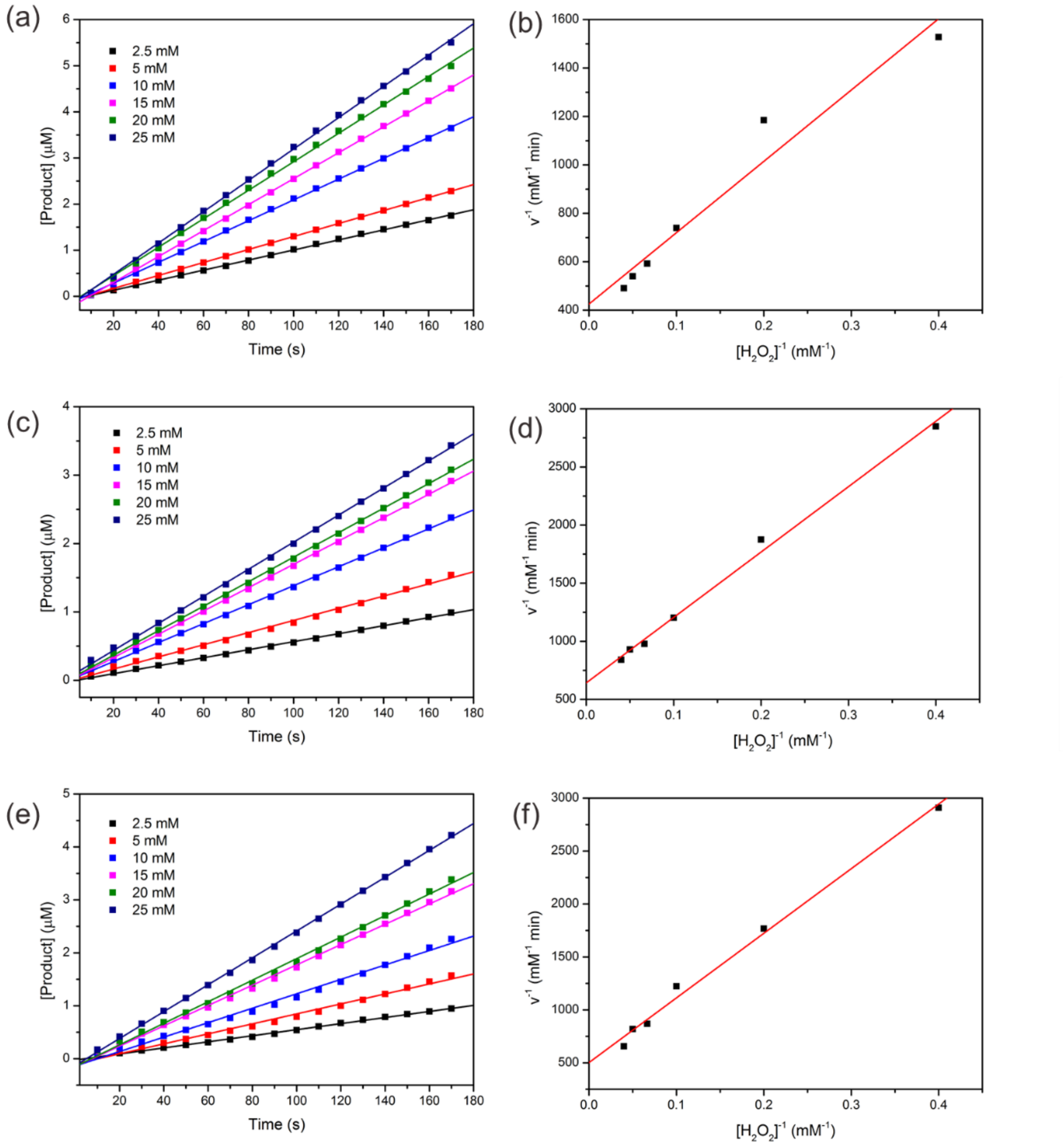

Supplementary Figure 21. The initial o-PDA oxidation profile catalyzed by free Cyt c (a), Cyt c@ CYCU-3 (c) and Cyt c@ CYCU-3D (e). The concentrations of H_2O_2 range from 2.5 mM to 25 mM. Lineweaver-Burk plot of o-PDA oxidation catalyzed by free Cyt c (b), Cyt c@ CYCU-3 (d) and Cyt c@ CYCU-3D (f).

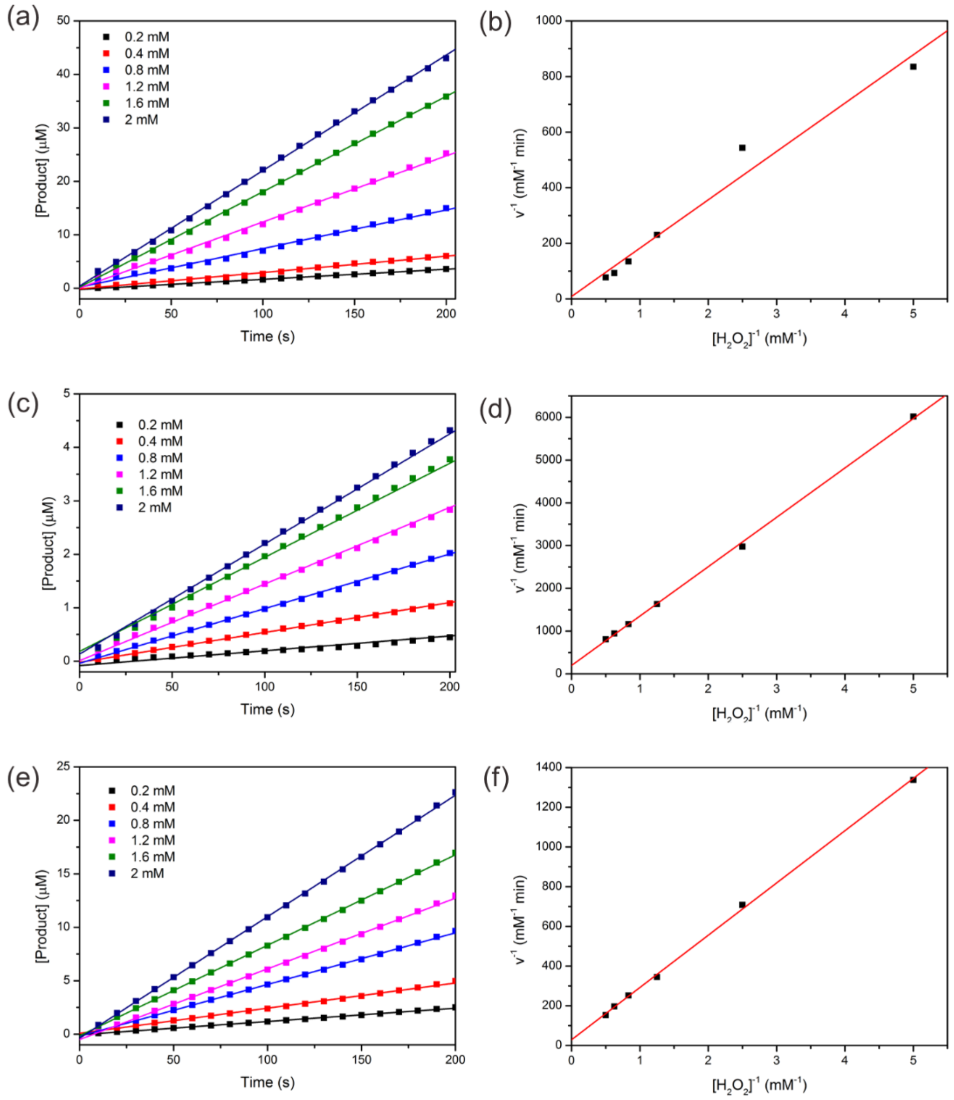

Supplementary Figure 22. The initial ABTS oxidation profile catalyzed by free Cyt c (a), Cyt c@ CYCU-3 (c) and Cyt c@ CYCU-3D (e). The concentrations of H_2O_2 range from 0.2 mM to 2 mM. Lineweaver-Burk plot of ABTS oxidation catalyzed by free Cyt c (b), Cyt c@ CYCU-3 (d) and Cyt c@ CYCU-3D (f).

Supplementary Table 1. Crystal data and structure refinements.

Name	PCN-160	PCN-160-100%	PCN-160-31%	PCN-160-31%-2
CCDC	1525246	1525247	1525248	1525249
Empirical formula	$C_{336}H_{192}N_{48}O_{128}Zr_{24}$	$C_{360}H_{216}N_{24}O_{128}Zr_{24}$	$C_{344}H_{200}N_{40}O_{128}Zr_{24}$	$C_{256}H_{148}N_{32}O_{128}Zr_{24}$
	$[Zr_6O_8]_4$ $[(C_{14}H_8O_4N_2)_{24}]$	$[Zr_6O_8]_4$ $[(C_{15}H_9O_4N)_{24}]$	$[Zr_6O_8]_4$ $[(C_{14}H_8O_4N_2)_{16}]$ $[(C_{15}H_9O_4N)_8]$	$[Zr_6O_8]_4$ $[(C_{14}H_8O_4N_2)_{14}]$ $[(C_{15}H_9O_4N)_4][O_{24}]$
Formula weight	9138.66	9114.85	9130.72	7909.34
Color/Shape	Orange octahedron	Yellow octahedron	Orange octahedron	Orange octahedron
Temperature/K	110(2)	100(2)	110(2)	103(2)
Crystal system	Cubic	Cubic	Cubic	Cubic
Space group	Fm-3m	Fm-3m	Fm-3m	Fm-3m
a/Å	29.4701(10)	29.3907(11)	29.5566(9)	29.590(10)
b/Å	29.470	29.391	29.557	29.590
c/Å	29.470	29.391	29.557	29.590
α/ °	90.00	90.00	90.00	90.00
β/ °	90.00	90.00	90.00	90.00
γ/ °	90.00	90.00	90.00	90.00
Volume/$Å^3$	25594.4(9)	25388.1(10)	25820.4(8)	25907(9)
Z	1	1	1	1
ρ_{calc} g/cm^3	0.593	0.596	0.587	0.507
μ/mm^{-1}	0.267	0.269	0.265	0.259
F(000)	4528.0	4528.0	4528.0	3892.0
Wavelength (Å)	0.71073 (MoKα)	0.71073 (MoKα)	0.71073 (MoKα)	0.71073 (MoKα)
2Θ range for data collection/°	4.58 to 54.96	4.6 to 54.96	4.58 to 54.84	4.56 to 54.78
Completeness	100%	100%	100%	100%
Reflections collected	34837	102552	63472	62181
Independent reflections	1513	1499	1518	1518
R_{int}	0.0454	0.0905	0.0571	0.0663
Data	1513	1499	1518	1518
Restraints	8	8	8	8
Parameters	27	30	30	33
GOF on F^2	1.074	1.740	1.061	1.110
R_1 [$I > 2\sigma(I)$][a]	0.0663	0.1308	0.0731	0.1097
wR_2 [$I > 2\sigma(I)$][b]	0.1794	0.2904	0.2017	0.3090
R_1 (all data)[a]	0.0722	0.1390	0.0777	0.1166
wR_2 (all data)[b]	0.1842	0.2942	0.2071	0.3156
Residue peak / hole (e$Å^{-3}$)	1.51/-1.01	1.17/-0.68	1.25/-0.96	2.04/-1.33

[a] $R_1 = \Sigma||F_o| - |F_c||/\Sigma|F_o|$.

[b] $wR_2 = |\Sigma w(|F_o|^2 - |F_c|^2)|/\Sigma|w(F_o^2)^2|^{1/2}$.

Supplementary Table 2. The exchange ratio in MOFs calculated by UV-vis and ^{1}H-NMR spectra, respectively.

CBAB concentration (mM)	0	5	10	20	30	40	80×5
**Exchange ratio by UV**	0	9%	17%	31%	43%	65%	100%
**Exchange ratio by ^{1}H-NMR**	0	8%	16%	27%	39%	59%	100%

Supplementary Table 3. Maximum pore size (Å) of PCN-160-R%-*C* calculated by DFT method.

**Exchange ratio (%)**	**0**	**9%**	**17%**	**31%**	**43%**
**0 AcOH**	13.6	13.6	14.8	14.8	14.8
**0.5 M AcOH**	14.8	25.1	25.1	25.1	54.3
**1 M AcOH**	14.8	25.1	58.7	93.1	137
**2 M AcOH**	14.8	68.0	108	137	186

Supplementary Table 4. Composition and CN of cluster of PCN-160-R%-*C*.

**Exchange ratio**	C_{AcOH}	**Composition**	**CN of Cluster**
**0**	**0**	$Zr_{12}O_8(OH)_8$ $AZDC_{11.5}\cdot(H_2O)(OH)$	11.5
**0**	**0.5 M**	$Zr_{12}O_8(OH)_8$ $AZDC_{11.0}\cdot(H_2O)_2(OH)_2$	11.0
**0**	**1 M**	$Zr_{12}O_8(OH)_8$ $AZDC_{11.2}\cdot(H_2O)_{1.6}(OH)_{1.6}$	11.2
**0**	**2 M**	$Zr_{12}O_8(OH)_8$ $AZDC_{11.5}\cdot(H_2O)(OH)$	11.5
**31%**	**0**	$Zr_{12}O_8(OH)_8$ $AZDC_{8.0}$ $CBAB_{3.6}\cdot(H_2O)_{0.8}(OH)_{0.8}$	11.6
**31%**	**0.5 M**	$Zr_{12}O_8(OH)_8$ $AZDC_{7.1}$ $CBAB_{2.1}\cdot(H_2O)_{5.6}(OH)_{5.6}$	9.2
**31%**	**1 M**	$Zr_{12}O_8(OH)_8$ $AZDC_{7.1}$ $CBAB_{1.8}\cdot(H_2O)_{6.2}(OH)_{6.2}$	8.9
**31%**	**2 M**	$Zr_{12}O_8(OH)_8$ $AZDC_{7.3}CBAB_{1.6}\cdot(H_2O)_{6.2}(OH)_{6.2}$	8.9

Supplementary Table 5. Comparison of kinetic parameters for Cyt c, Cyt c@ CYCU-3 and Cyt c@ CYCU-3D.

**Catalyst**	**Substrate**	**K_m (mM)**	**k_{cat} (min)**	**k_{cat}/K_m**	**V_{max} (mM/min)**
Cyt c	o-PDA	6.92	2.13	0.308	0.00106
Cyt c@ CYCU-3D	o-PDA	12.1	1.81	0.148	0.000905
Cyt c@ CYCU-3	o-PDA	8.75	1.41	0.161	0.000707
Cyt c	ABTS	19.9	104	5.22	0.0521
Cyt c@ CYCU-3D	ABTS	9.05	31.2	3.45	0.0156
Cyt c@ CYCU-3	ABTS	5.87	4.62	0.787	0.00231

Supplementary Methods

Synthesis of H_2CBAB (4-carboxybenzylidene-4-aminobenzoic acid). In separate beakers, 4-amino benzoic acid (1.4 g, 10 mmol) and 4-formylbenzoic acid (1.5 g, 10 mmol) were dissolved respectively in 50 mL methanol. The solutions were mixed in a 500 mL flask and refluxed for 3 h under continuous stir. After cooling to room temperature, the precipitates were collected by filtration and washed with methanol (2.5 g, yield: 92%). ^{1}H-NMR (300 MHz, DMSO-d_6) δ 8.74 (s, 1H), 8.08 (d, 4H), 8.00 (m, 2H), 7.36 (m, 2H).

Synthesis of H_2AZDC (azobenzene-4,4'-dicarboxylic acid). The H_2AZDC was synthesized according to the literature with slight modification.[1] In a 500 mL flask, p-nitrobenzoic acid (11 g, 66 mmol) and sodium hydroxide (40 g, 100 mmol) were dissolved in 150 ml of water. The temperature of the mixture was controlled at 50 ℃ in a water bath. A solution of glucose (90 g in 100 ml of water) was added dropwise over 60 min. The mixture was air bubbled under continuous stir overnight and 50 mL methanol was added. The precipitates were collected by filtration, washed with methanol and dissolved in hot water. The solution was acidified with 10% HCl aqueous solution and the product was collected by filtration (6.1 g, yield: 68%). ^{1}H-NMR (300 MHz, DMSO-d_6) δ 8.17 (m, 4H), 8.02 (m, 4H).

Synthesis of PCN-160. $ZrCl_4$ (200 mg), H_2AZDC (100 mg), trifluoroacetic acid (1.0 mL) and DMF (20 mL) were charged in a Pyrex vial. The mixture was heated in 120 ℃ oven for 72 h. After cooling down to room temperature, the red crystals were harvested (95 mg, yield: 67%).

Synthesis of PCN-160-R%. PCN-160-R% (R=6, 9, 17, 31, 43, and 65) were synthesized by the linker exchange of PCN-160 with CBAB solution of different concentration (2.5, 5, 10, 20, 40, and 80 mM, respectively). Generally, crystals of PCN-160 (100 mg) were incubated with the solution of H_2CBAB in DMF (20 mL) at 75 ℃ for 10 h. PCN-160-100% were synthesized by repeatedly exchange the supernatant with fresh CBAB stock solution (80 mM) every 5 h for 4 times. The crystals of PCN-160-R% were collected by filtration and washed with fresh DMF 3 times.

Synthesis of PCN-160-R%-*C*. PCN-160-R%-*C* (*C* = 0.5, 1, and 2) was synthesized by treating the crystals of PCN-160-R% with different concentration of AcOH in DMF solution (0.5, 1, and 2 M, respectively). Generally, crystals of PCN-160-R% (100 mg) were incubated with the solution of AcOH in DMF (20 mL) at 40 ℃ for 24 h. The crystals were collected by filtration and washed with fresh DMF and water to

remove any remaining acetic acid.

Synthesis of CYCU-3. $AlCl_3$ (75 mg), H_2AZDC (150 mg), acetic acid (0.15 mL) and DMF (15 mL) were charged in a Pyrex vial. The mixture was heated in 120 ℃ oven for 24 h. After cooling down to room temperature, the yellow crystalline powders were harvested (65 mg, yield: 37%).

Synthesis of CYCU-3-22%. $AlCl_3$ (75 mg), H_2AZDC (100 mg), H_2CBAB (50 mg), acetic acid (0.15 mL) and DMF (15 mL) were charged in a Pyrex vial. The mixture was heated in 120 ℃ oven for 24 h. After cooling down to room temperature, the yellow crystalline powders were harvested (55 mg, yield: 32%). The CBAB in the product is 22% determined by UV-vis spectroscopy.

Synthesis of CYCU-3-22%-0.1. CYCU-3-22% (100 mg) were incubated with the solution of HCl in DMF (0.1 mM, 20 mL) at 40 ℃ for 24 h. The products were collected by filtration and washed with fresh DMF and water to remove any remaining acid.

Synthesis of CYCU-3-22%-0.2. CYCU-3-22% (100 mg) were incubated with the solution of HCl in DMF (0.2 mM, 20 mL) at 40 ℃ for 24 h. The products were collected by filtration and washed with fresh DMF and water to remove any remaining acid.

Single Crystal X-ray Crystallography. All crystals were taken from the mother liquid without further treatment, transferred to oil and mounted into a loop for single crystal X-ray data collection. Diffraction was measured on a Bruker Venture CMOS diffractometer equipped with a Cu-K_α sealed-tube X-ray source (λ = 1.5406 Å). The data frames were recorded using the program APEX2 and processed using the Apex3 suite of programs.[2] The data were corrected for absorption and beam corrections based on the multi-scan technique as implemented in SADABS. The structures were solved by direct method using SHELXS and refined by full-matrix least-squares on F^2 using SHELXL software.[3]

Refinement Details of PCN-160. Diffraction frames were integrated in Cubic F by APEX2 and determined with the R(sym) of 0.051 by *XPREP*. *XPREP* suggested that $Fm\bar{3}m$ be the best centrosymmetric space group (COM=4.81). The AZDC ligand is two-fold disordered in the structure. By careful restraints of the ligand geometry and the atomic displacement parameters, $R1$ value is 0.1069 before *SQUEEZE* treatment and 0.0663 after *SQUEEZE* treatment. The squeezed void volume was 18776 Å^3, equivalent to 73.4% of the unit cell.

Refinement Details of PCN-160-100%. Diffraction frames were integrated in Cubic

F by APEX2. Based on our previous experience on the isostructural PCN-160, $Fm\bar{3}m$ was selected to describe the structure. The CBAB ligand is two-fold disordered in the structure. By careful restraints of the ligand geometry and the atomic displacement parameters, $R1$ value is 0.1347 before *SQUEEZE* treatment and 0.1308 after *SQUEEZE* treatment. The squeezed void volume was 18430 Å^3, equivalent to 72.6% of the unit cell.

Refinement Details of PCN-160-31%. Diffraction frames were integrated in Cubic F by APEX2. Based on our previous experience on the isostructural PCN-160, $Fm\bar{3}m$ was selected to describe the structure. According to the UV-vis results, this structure contains both AZDC (~66. 67%) and CBAB ligand (~33.33%). The each ligand is two-fold disordered in the structure. By careful restraints of the ligand geometry and the atomic displacement parameters, $R1$ value is 0.1085 before *SQUEEZE* treatment and 0.0731 after *SQUEEZE* treatment. The squeezed void volume was 18912 Å^3, equivalent to 73.2% of the unit cell.

Refinement Details of PCN-160-31%-2. Diffraction frames were integrated in Cubic F by APEX2. Based on our previous experience on the isostructural PCN-160, $Fm\bar{3}m$ was selected to describe the structure. According to the UV-vis results, this structure contains both AZDC (77.78%) and CBAB ligand (22.22%). The each ligand is two-fold disordered in the structure. The occupancy of the ligand is refined as 75% and the occupancy of terminal -OH/H_2O is refined as 25%, corresponding to the experimental result that about 25% of the ligand is replaced by terminal -OH/H_2O as crystal defects. By careful restraints of the ligand geometry and the atomic displacement parameters, $R1$ value is 0.1097 before *SQUEEZE* treatment.

Determination of Exchange Ratio. No weight loss was observed for MOF samples during linker exchange process suggesting that linker exchange happen without dissolving or destruction of framework. Therefore, the moles of AZDC in the solution equals to the moles of CBAB being exchanged into the framework. The exchange ratio in MOFs (R%), defined as moles of CBAB divided by the moles of organic linkers (i.e. CBAB+AZDC), was calculated from AZDC concentration in solution determined by UV-vis spectra.

$$R\% = \frac{\mathrm{MW_{AZDC}} \cdot \mathrm{C_{AZDC}} \cdot \mathrm{V_{solution}}}{\mathrm{m_{MOF}} \cdot 69\%}$$

^{1}H NMR spectroscopy. The exchange ratio in MOFs were further confirmed by the ^{1}H-NMR results of digested samples (Supplementary Table 2). For ^{1}H NMR spectroscopy, the dry samples (around 5 mg) were dissolved in about 1 mL 5% D_2SO_4/DMSO-d_6. The CBAB was decomposed into 4-amino benzoic acid and

4-formylbenzoic acid under acetic condition. Therefore, the exchange ratio were calculated by the integration of H from AZDC and 4-formylbenzoic acid.

Gas Sorption Measurements. Gas sorption measurements were conducted using a Micrometritics ASAP 2020 system. Before gas sorption experiment, MOF samples was washed with DMF and water, and exchanged by acetone for 3 days, during which the solvent was decanted and freshly replenished three times. The solvent was removed under vacuum at 100 ^{o}C for 10 h, yielding porous material.

Defect Analysis. We proposed that labile CBAB linker is partially dissociated and removed during acid treatment to create missing-linker defects. Although the exact identity of defect sites is unknown, most likely, missing linkers would be replaced with $–OH_2$ and –OH groups in such a way as to compensate for the charge loss from the linkers. The weight percent of CBAB and AZDC in PCN-160-31%-*C* treated by different concentration of AcOH were analyzed by UV-vis spectra. About 50 mg PCN-160-31%-*C* samples were precisely weighted and dissolved in 50 mL K_2CO_3 aqueous solution (5%) to get a clear solution. The concentration of AZDC and CBAB were analyzed by the comparison of the UV-vis absorbance (at λ = 432 and 266 nm, respectively). Note that the CBAB was decomposed into 4-amino benzoate and 4-formylbenzoate in K_2CO_3 aqueous solution. The concentration of CBAB was analyzed by measuring the UV-vis absorbance of 4-amino benzoate. The composition and connection number (CN) of Zr_6 cluster of each sample are calculated according to the weight percent of CBAB and AZDC (Supplementary Table 4).

The concentration of CBAB and AZDC in the solution after linker labilization process were also analyzed by UV-vis spectra. Crystals of PCN-160-R% (~100 mg) were incubated with the solution of AcOH in DMF (20 mL) at 40 ℃ for 24 h. 0.1 mL of solution was sampled and added with 1.9 mL K_2CO_3 aqueous solution for UV-vis absorbance measurements. The concentration of CBAB is much higher than that of AZDC, indicating the exclusive removal of labile CBAB linker instead of AZDC linker. (Supplementary Figure 8) Meantime, the appearance of Zr^{4+} in the solution determined by inductively coupled plasma mass spectrometry (ICP-MS) suggests the missing-cluster defects.

Thermogravimetric Analysis. For thermogravimetric analysis, about 10 mg of the sample was heated on a TGA-50 (SHIMADZU) thermogravimetric analyzer from room temperature to 700 ℃ at a rate of 5 ℃ min^{-1} under N_2 flow of 15 mL min^{-1}. The initial weight loss before 300 ℃ is attributed to the removal of the coordinated water molecules on the clusters, which is comparable to the theoretical mass percentage.

TGA curves indicate thermal stability of the framework up to 450 ℃, where decomposition of the framework starts.

Molecular Modeling. The missing-linker model was built by taking a stoichiometric 3 × 3 × 3 supercell of PCN-160 with face-centered cubic (**fcu**) topology and randomly removing a certain amount of linkers. We developed a customized perl script in Material Studio package to randomly delete the ligands in 3 × 3 × 3 supercell of PCN-160. The 3 × 3 × 3 supercell was chosen because it is the largest model Zeo++ package could handle.[4] API (Application Program Interface) of Material Studio was adopted to manipulate the atom, bond and fragment objects. Firstly, the ligands were detached from metal cluster by deleting the O-C bonds. Then a search for N-N bonds was made so that we can identify all the ligand fragments in the supercell. Later, a random number list was generated based on the deleting percentage of ligands. The randomly selected ligand fragments were deleted. Finally, the O-C bonds between metal cluster and ligands were added back into the supercell to restore the bonds connectivity in the system. The hydrogen on the terminal -OH/H_2O ligands were automatically added by Material Studio. Similarly, a script for metal cluster random deletion was developed. However, for metal cluster deletion, the ligands around the metal cluster were also deleted based on the observation that terminal carboxylates could be removed under acidic condition. The major part of these scripts was listed as follow:

```
#!perl
use strict;
use Getopt::Long;
use MaterialsScript qw(:all);
my $doc = $Documents{"filename.xsd"};
my $NumberOfDeleted=48; #input: number of deleted ligands.
my $bonds = $doc->UnitCell->Bonds;
our @fragments=();
our $fragmentsnum=0;
foreach my $bond (@$bonds) {
if (($bond->Atom1->ElementSymbol eq "N") and   ($bond->Atom2->ElementSymbol eq "N")){
    my $fragmentAtoms=$bond->Atom1->Fragment->Atoms;
    $fragmentsnum=$fragmentsnum+1;
    push(@fragments,[($fragmentAtoms,$fragmentsnum)]);
 }
}
my $range = $fragmentsnum;
# building deleting list
our @randnumlist=();
our $randnumconter=0;
do{  my $random_number = int(rand($range));
     my $flag=0;
   foreach my $j(@randnumlist){
               if($random_number eq $j){$flag=1;}
            }
   if($flag eq "0"){
push(@randnumlist,$random_number);
$randnumconter=$randnumconter+1;
}
}while( $randnumconter< $NumberOfDeleted );
#check the length of randnumlist
my $ii=0;
foreach my $i (@randnumlist) {
$ii=$ii+1;
}
if ($ii eq $NumberOfDeleted){
    #delete the ligand
    foreach my $i (@randnumlist) {
        @fragments[$i]->[0]->Delete;
        print "Deleting ligand NO.:",$i,"\n";
      }
}
print "fragments number is:###",$fragmentsnum,"###","\n";
print "% of ligands had been deleted:",100*$NumberOfDeleted/$fragmentsnum,"%","\n";
#calculate bonds
$doc->CalculateBonds;
```

Based on the script above, we developed two types of defect models, namely, a missing-linker model and a missing-cluster model. The pore size distributions were simulated by the Zeo++ package.[4] The simulated pore distribution for missing cluster model provides clear evidence that the experimentally observed mesopores originated from missing-cluster defects. The missing-linker defects alone will not lead to the formation of 2.5 nm mesopores. Therefore, the 2.5 nm mesopores are tentatively attributed to the missing-cluster defects, in which a Zr_6 cluster is removed along with linkers around it. As the missing-cluster defects locally created an eight-connected net with ReO_3 (**reo**) topology, it is also refered as **reo** defects.

$Cr_2O_7^{2-}$ Adsorption. About 50 mg of dry samples were precisely weighted and used for the removal of dichromate with a concentration of 100 ppm. The dichromate aqueous solutions (20 mL) containing the adsorbents were mixed well with magnetic stirring and maintained for a certain time (from 1 to 60 min) at 25 ℃. 0.1 mL of solution were sampled each time, diluted into 2 mL, and then separated from the adsorbent with a syringe filter (PTFE, 0.25 μm). The dichromate concentrations were calculated by the comparison of the UV-vis absorbance (at λ = 257 nm).

POM Adsorption. $Na_6K_4[Ni_4(H_2O)_2(PW_9O_{34})_2]\cdot 32H_2O$ was synthesized according to the literature.[5] About 50 mg of dry samples were precisely weighted and mixed well with 10 mL of $H_3PMo_{12}O_{40}$ in methanol/H_2O (0.25%) under magnetic stirring at 25 ℃. 0.1 mL of solution were sampled each time, diluted into 2 mL, and then separated from the adsorbent with a syringe filter (PTFE, 0.25 μm). The POM concentration were calculated by the comparison of the UV-vis absorbance (at λ = 250 nm).

MOP Adsorption. $Cu_{24}(BDC\text{-}OH)_{24}(H_2O)_{24}$ (MOP) was synthesized according to the literature.[6] About 50 mg of dry samples were precisely weighted and mixed well with 10 mL of MOP in DMF solution (1%) under magnetic stirring at 25 ℃. 0.1 mL of solution were sampled each time, diluted into 2 mL, and then separated from the adsorbent with a syringe filter (PTFE, 0.25 μm). The MOP concentration were calculated by the comparison of the UV-vis absorbance (at λ = 690 nm).

Creating defects in CYCU-3. Labile CBAB linkers were doped in CYCU-3 during the synthesis by using a mixture of CBAB (50mg) and AZDC (100mg) as starting materials. The linker ratio of CBAB in the product is 22%, which was determined by UV-vis spectroscopy. The samples with 22% of labile CBAB linkers was denoted as CYCU-3-22%. The crystallinity and the porosity of samples are well maintained after the doping of CBAB as proved by PXRD patterns and N_2 sorption isotherms

(Supplementary Figure 18, 19).

Defects were created in CYCU-3-22% by treating with the solution of HCl in DMF at 40 ℃ for 24 h. The crystallinity of the samples are maintained after acid treatment, which is confirmed by PXRD patterns (Supplementary Figure 18). N_2 sorption isotherms and pore size distributions show the growth of mesopores with a diameter of ~7 nm along with the decrease of small mesopore volume (3 nm in diameter). This indicates that the 3 nm mesopores were gradually extended to the ~7 nm large mesopores by acid etching. CYCU-3-22%-0.2 shows high porosity and large mesopores so that it is used for enzyme encapsulation and catalysis studies.

Enzyme loading. The cytochrome c (Cyt c) solution (300 μM) were incubated with activated CYCU-3-22% and CYCU-3-22%-0.2 (10 mg) at 25 ℃. The resulted enzyme loaded CYCU-3-22% and CYCU-3-22%-0.2 were denoted as Cyt c@ CYCU-3 and Cyt c@ CYCU-3D respectively where D stands for defected samples. The uptake of Cyt c was determined by UV-visible spectroscopy. The absorption of the soret band at 399 nm was proportional to the concentration of enzyme according to the Beer-Lambert Law. The loading amount of enzymes was calculated by measuring the concentration of enzymes in the supernatant and then subtracting from the free-enzyme amount added in the beginning of experiment. The enzyme uptake reached the saturation in 48 h since no more change on the soret band of supernatant was observed. The maximum loadings are 23.3 mmol/g for Cyt c@ CYCU-3 and 14.5 mmol/g for Cyt c@ CYCU-3D. Before catalytic reaction, the solid samples were immersed in water for two days during which the solvent was decanted and freshly replenished five times to ensure full removal of any loosely bound Cyt c. N_2 sorption isotherms of CYCU-3 and CYCU-3D after enzyme loading prove that the enzyme occupies the hexagonal channels, leaving the triangular channels poised for substrates.

Kinetic studies. Cyt c can catalyze the oxidation of o-phenylenediamine (o-PDA) and 2-2'- azino-bis(3-ethylbenzthiazoline-6-sulfonic acid) (ABTS) in the presence of H_2O_2. The products were confirmed by scanning the UV-vis absorbance on spectrophotometer and the concentrations of products were calculated by their molar extinction coefficients at respective wavelengths (16300 $M^{-1}cm^{-1}$ at 450 nm for the peroxidized product of o-PDA and 3.6×104 $M^{-1}cm^{-1}$ at 418 nm for peroxidized product of ABTS). The concentration of H_2O_2 was determined spectrophotometrically at 240 nm with ε = 43.6 $M^{-1}cm^{-1}$. Kinetic measurements were carried out in time course mode by monitoring the absorbance change at 450 nm for o-PDA and 418 nm for ABTS. The Michealis-Menten equation was used for enzyme kinetics.

$$V_0 = \frac{V_{max}[S]}{K_m + [S]}$$

V_0 is the initial catalytic rate, V_{max} is the maximum reaction rate, which is obtained when the catalytic sites on the enzyme are saturated with substrate. [S] is the substrate concentration and K_m is the apparent Michealis-Menten constant. Lineweaver-Burk plot was employed for illustrating kinetic data and calculate the parameters by taking the reciprocal of both sides of the Michaelis–Menten equation.

The reactions were conducted in the 3ml cuvette directly. The substrate (2 mM) and Cyt c (or MOF immobilized Cyt c, 0.5 μM) were added to buffer followed with the addition of H_2O_2. After a quick shaking, the cuvette was put in the UV sample holder as quickly as possible. The data were collected using the kinetic mode and the plots of absorbance to time were obtained. The background need to be scanned before data collection with addition of water instead of H_2O_2. The specific activities for Cyt c, Cyt c@ CYCU-3 and Cyt c@ CYCU-3D were measured with 2 mM ABTS, 20 mM H_2O_2, 0.5 μM enzyme in 3 ml water at 25 ℃. The specific activities were calculated by comparing the reaction rate catalyzed by each catalysts.

Supplementary References

1. Li, J., Cvrtila, I., Colomb-Delsuc, M., Otten, E. & Otto, S. An “ingredients” approach to functional self-synthesizing materials: A metal-ion-selective, multi-responsive, self-assembled hydrogel. *Chem. Eur. J.* **20**, 15709-15714 (2014).
2. APEX2 v2012.2.0 and SAINT v7.68A data collection and data processing programs, respectively. Bruker Analytical X-ray Instruments, Inc., Madison, WI; SADABS v2008/1 semi-empirical absorption and beam correction program. Sheldrick, G. M. University of Göttingen, Germany.
3. Sheldrick, G. M. *SHELXTL*, Version 6.14, Structure Determination Software Suite, Bruker AXS, Madison, WI, 2003.
4. Willems, T. F., Rycroft, C. H., Kazi, M., Meza, J. C. & Haranczyk, M. Algorithms and tools for high-throughput geometry-based analysis of crystalline porous materials. *Micropor. Mesopor. Mater.* **149**, 134-141 (2012).
5. Lv, H. *et al.* A noble-metal-free, tetra-nickel polyoxotungstate catalyst for efficient photocatalytic hydrogen evolution. *J. Am. Chem. Soc.* **136**, 14015-14018 (2014).
6. Li, J.-R. & Zhou, H.-C. Bridging-ligand-substitution strategy for the preparation of metal–organic polyhedra. *Nat. Chem.* **2**, 893-898 (2010).
